# Intestinal Preservation Injury: A Comparison Between Rat, Porcine and Human Intestines

**DOI:** 10.3390/ijms20133135

**Published:** 2019-06-27

**Authors:** John Mackay Søfteland, Anna Casselbrant, Ali-Reza Biglarnia, Johan Linders, Mats Hellström, Antonio Pesce, Arvind Manikantan Padma, Lucian Petru Jiga, Bogdan Hoinoiu, Mihai Ionac, Mihai Oltean

**Affiliations:** 1 The Transplant Institute, Sahlgrenska University Hospital, 413 45 Gothenburg, Sweden; 2 Laboratory for Transplantation and Regenerative Medicine, Institute of Clinical Sciences, Sahlgrenska Academy at the University of Gothenburg, Sahlgrenska Science Park Medicinaregatan 8, 413 90 Gothenburg, Sweden; 3 Department of Gastrosurgical Research and Education, Institute of Clinical Sciences, Sahlgrenska Academy at the University of Gothenburg, Sahlgrenska University Hospital, 41345 Gothenburg, Sweden; 4 Department of Transplantation, Skåne University Hospital, 205 02 Malmö, Sweden; 5 Department of Medical and Surgical Sciences and Advanced Technologies, University of Catania, Via Santa Sofia 86, 95123 Catania, Italy; 6 Department for Plastic, Aesthetic, Reconstructive and Hand Surgery, Evangelisches Krankenhaus Oldenburg, Medical Campus University of Oldenburg, Steinweg 13–17, 26122 Oldenburg, Germany; 7 Pius Branzeu Center for Laparoscopic Surgery and Microsurgery, University of Medicine and Pharmacy, P-ta. E. Murgu 2, 300041 Timisoara, Romania

**Keywords:** tight junctions, organ preservation, intestine, transplantation, ischemia, intestinal mucosa

## Abstract

Advanced preservation injury (PI) after intestinal transplantation has deleterious short- and long-term effects and constitutes a major research topic. Logistics and costs favor rodent studies, whereas clinical translation mandates studies in larger animals or using human material. Despite diverging reports, no direct comparison between the development of intestinal PI in rats, pigs, and humans is available. We compared the development of PI in rat, porcine, and human intestines. Intestinal procurement and cold storage (CS) using histidine–tryptophan–ketoglutarate solution was performed in rats, pigs, and humans. Tissue samples were obtained after 8, 14, and 24 h of CS), and PI was assessed morphologically and at the molecular level (cleaved caspase-3, zonula occludens, claudin-3 and 4, tricellulin, occludin, cytokeratin-8) using immunohistochemistry and Western blot. Intestinal PI developed slower in pigs compared to rats and humans. Tissue injury and apoptosis were significantly higher in rats. Tight junction proteins showed quantitative and qualitative changes differing between species. Significant interspecies differences exist between rats, pigs, and humans regarding intestinal PI progression at tissue and molecular levels. These differences should be taken into account both with regards to study design and the interpretation of findings when relating them to the clinical setting.

## 1. Introduction 

Intestinal transplantation is the established therapeutic alternative in patients with complicated intestinal failure, with results continuously improving over the last two decades [1]. However, the post-transplant course is frequently marred by life-threatening complications due to ischemia–reperfusion injury (IRI), immunosuppression, and acute rejection, and patient management remains challenging [2,3,4]. Hence, further translational research is warranted to develop novel strategies to alleviate IRI, identify noninvasive biomarkers of rejection, and test alternative immunosuppressive strategies.

Intestinal grafts withstand the shortest cold storage (CS) period of all abdominal organs. In the clinical setting, CS is kept below ten hours due to concerns of mucosal sloughing and epithelial barrier breakdown, which may favor bacterial translocation and graft edema [5,6]. Numerous experimental approaches targeting the preservation injury have been tested in rats [7], but virtually none have been implemented clinically due to the lack of consistent evidence, including preclinical safety studies. 

Rats have the advantage of simpler logistics, lower costs, and a relatively straightforward surgical procedure. Rat models have provided valuable insights into the physiology, immunology, and pathology of the transplanted intestine [8,9]. Nonetheless, anatomical, physiological, and immunological differences prevent the direct translation of many findings into clinical practice, and pigs are frequently used as a preclinical model to confirm the results of small animal studies [10,11,12,13]. Pigs share numerous anatomical and physiological similarities with humans, are easily accessible and affordable, and their use as livestock animals relieves some ethical concerns. 

In spite of their use in intestinal preservation research, no direct comparison exists between rat and porcine intestines, to link the abundant data from rodents with this important preclinical model. To our knowledge, the extent to which the results obtained using porcine or rat intestines apply to the human intestine also remains unclear. Hence, it is unclear if and how the sequence and speed of development of the cellular and molecular alterations in rodents resemble the ones described in pigs and how this wealth of experimental data ultimately compares to the clinical setting. In this study, we set out to compare the development of the intestinal preservation injury in rats, pigs, and humans under similar conditions of procurement and CS. 

## 2. Results

### 2.1. Histology

**Figure 1:**
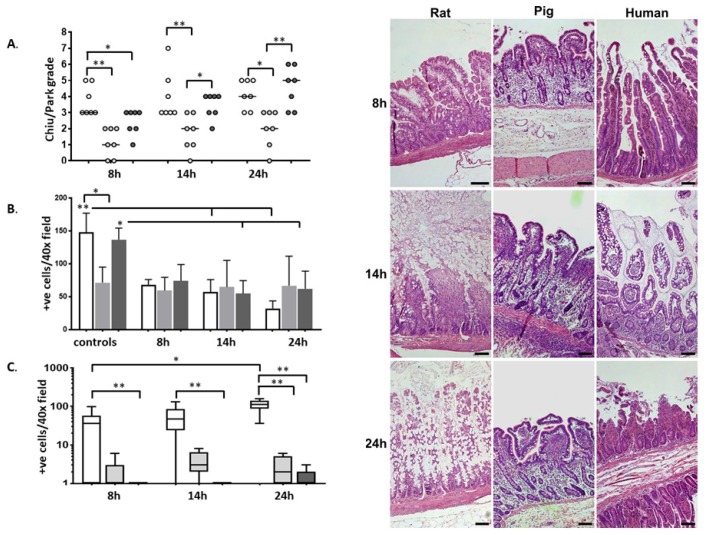
Light microscopy of rat (white), pig (light grey), and human (dark grey) intestines after different periods of cold storage (CS). (**A**) Summary of the tissue injury (Chiu score) induced by CS with each dot representing one individual (*n* = 7) and the bar showing the median value; (**B**) goblet cell count; (**C**) enterocyte apoptosis quantified by caspase-3 positive cells (box plot showing the median, 5–95th percentile, and lowest and highest values at each time point). * *p* < 0.05, ** *p* < 0.01. A large number of apoptotic enterocytes (positive for active caspase-3) were found in rat intestines after 8 h of CS. Rat intestines had more apoptotic enterocytes than human intestines at all time points (Figure 1C). Right: representative microphotographs from each species at each of the three time-points (hematoxillin eosin stain, original magnification ×100, scale bar 100 microns).

Intestinal CS induced the typical subepithelial lifting and edema in the vast majority of samples irrespective of species but the extent and speed of development of the subepithelial cleft revealed differences between species. Rat intestines developed significant subepithelial edema and even epithelial shedding (median Chiu/Park score 3) already after eight hours of CS, whereas the porcine intestines showed significantly lower injury score and mild edema or even normal histology (median Chiu/Park score 1) (*p* < 0.001). At the same time-point, human intestines exhibited mild or moderate subepithelial edema (median score 3)—a lesser injury than in rats (*p* = 0.04), but higher than in pigs (*p* = 0.02) (Figure 1A). 

At both latter time-points, rat intestines showed a significantly more severe mucosal injury compared to porcine intestines; human intestines revealed significantly worse morphology compared to pigs after 24 h (*p* < 0.01). A particular feature in the porcine and human intestines was the significant lifting of the mucosa from the muscular layer (submucosal edema), a feature not present in the rat intestines. 

Throughout the study, goblet cell (GC) counts indicated different patterns between the three species. Control rat intestine had significantly more GC than pig ileum (148 ± 28 vs 71 ± 23, *p* < 0.05). In rat and human intestines, CS induced a decrease in mucus-filled GC on the villi, which became significant after 14 h, whereas the amount of GC in porcine intestines did not differ significantly from the baseline throughout the entire experiment (Figure 1B). 

**Figure 2:**
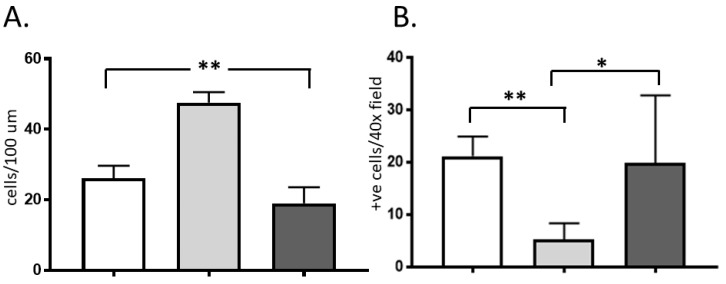
Enterocyte (**A**) and polymorphonuclear (PMN) leukocyte (**B**) counts in rat, (white bar) pig (light grey), and human (dark grey) intestines (*n* = 7). Enterocytes were counted using 4′,6-diamidino-2-phenylindole (DAPI) staining on the sides of longitudinally oriented villi on several 100 µm segments; PMNs were counted in villi on ten random fields at high magnification (×400) (data shown as mean ± SD). * *p* < 0.05, ** *p* < 0.01.

Normal pig intestines had significantly higher enterocyte density and significantly fewer polymorphonuclear neutrophils (PMN) in the villi compared to rat and human intestines (Figure 2).

### 2.2. Immunohistochemistry

In all three species, zonula occludens (ZO)-1 was detected as an intense, thin fluorescent signal at the apical tips of the basolateral membrane, from the crypts to the tip of the villi. Whereas ZO-1 staining in rats and humans frequently appeared like a line or large dots, ZO-1 staining in pigs often had the appearance of a dotted line or small dots (probably due to a narrower apical membrane and higher cellularity). Claudin-3 was visualized as a thin, reticular signal along the entire basolateral membrane. Claudin-3 frequently colocalized with ZO-1 in an area corresponding to the apical edge of the basolateral membrane (data not shown). 

**Figure 3:**
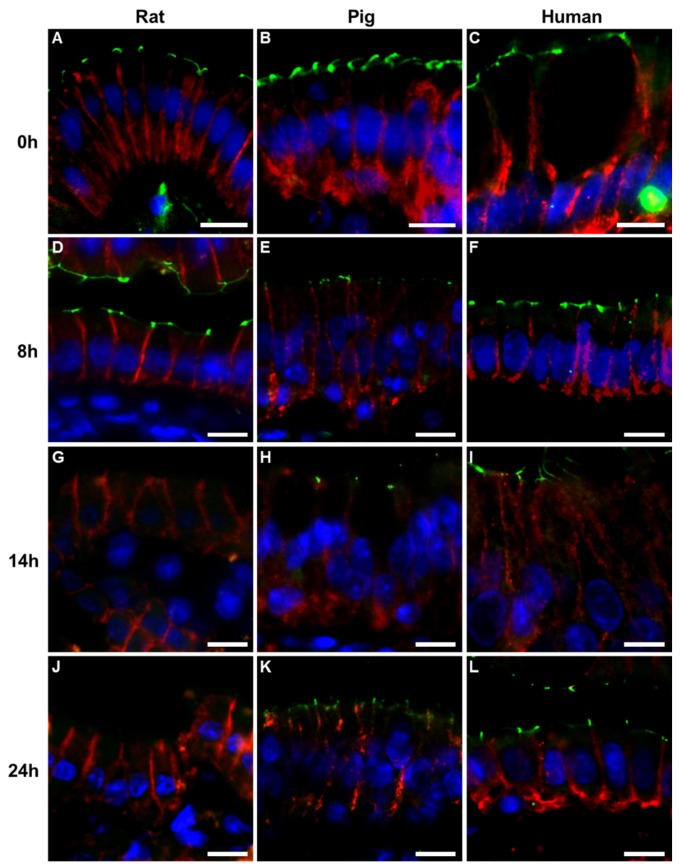
Immunofluorescent staining for zonula occludens (ZO)-1 (green) and claudin-3 (red) after various periods of cold storage (CS); strong immunofluorescent signal for both proteins after 8 h CS in rat (**A**), pig (**B**), and human intestine (**C**); after 14 h, ZO-1 signal was lost in rat (**D**) but not pig (**E**) or human (**F**) intestinal mucosa; after 24 h (**J**–**L**) of CS, ZO-1 staining was absent and claudin-3 revealed diffuse membrane staining and cytoplasmic staining in rat intestines (**G**), while in pig (**H**) and human (**I**) intestines, ZO-1 signal was maintained and claudin-3 stained more diffuse, stronger on the subjunctional basolateral membrane, together with some cytoplasmic staining. Nuclei were stained blue using 4′,6-diamidino-2-phenylindole (DAPI). Images were acquired from areas where enterocytes still remained attached to the lamina propria and as close to the villus tip as possible. Original magnification ×400, scale bar, 10 μm.

After eight hours of CS, ZO-1 staining became absent or discontinuous at the tip of some villi in rat intestines but overall it was maintained along the entire villus (Figure 3). Pig and human intestines revealed a strong immunosignal along the entire contour of the villi. In rats, claudin-3 staining was found between enterocytes but showed a widespread de-colocalization from ZO-1 as well as some cytoplasmic staining. Both pig and human showed strong claudin-3 staining as a sharp, reticular fluorescence signal along the entire basolateral membrane (Figure S1).

In rats, fourteen hours of CS led to a marked decrease in the ZO-1 immunostaining, which was preserved only towards the base of the villi and in the crypts. Porcine and human intestines continued, however, to show unchanged, well-preserved ZO-1 expression along the villus. Claudin-3 staining between enterocytes became more diffuse while cytoplasmic staining was also noted. Overall, the staining pattern remained thin and fibrillar but with a tendency towards less sharp, diffuse membrane staining and cytoplasmic staining. Stronger subjunctional intensity was also noted in some samples.

After 24 h, all rat intestines completely lacked villus staining for ZO-1, while both porcine and human continued to show immunofluorescent staining frequently reaching villus tips. In both pig and human intestines, claudin-3 revealed more diffuse, discontinuous staining along the basolateral membrane with an obvious subjunctional staining gradient. 

### 2.3. Western Blot Analysis

All proteins analyzed by Western blot were detected in rat, pig, and human samples. Generally, all proteins studied were found to have the lowest expression in the rat small intestine. After eight hours of CS, the expression of claudin-3, claudin-4, tricellulin, and ZO-1 was significantly higher in human samples compared to rat samples. This difference persisted after fourteen and 24 h for claudin-4 but subsided for claudin-3, tricellulin, and ZO-1. 

In four out of six tight junction (TJ) proteins studied (occludin, tricellulin, claudin-3, ZO-1) no differences between species were found after 14 h and 24 h of CS. 

**Figure 4:**
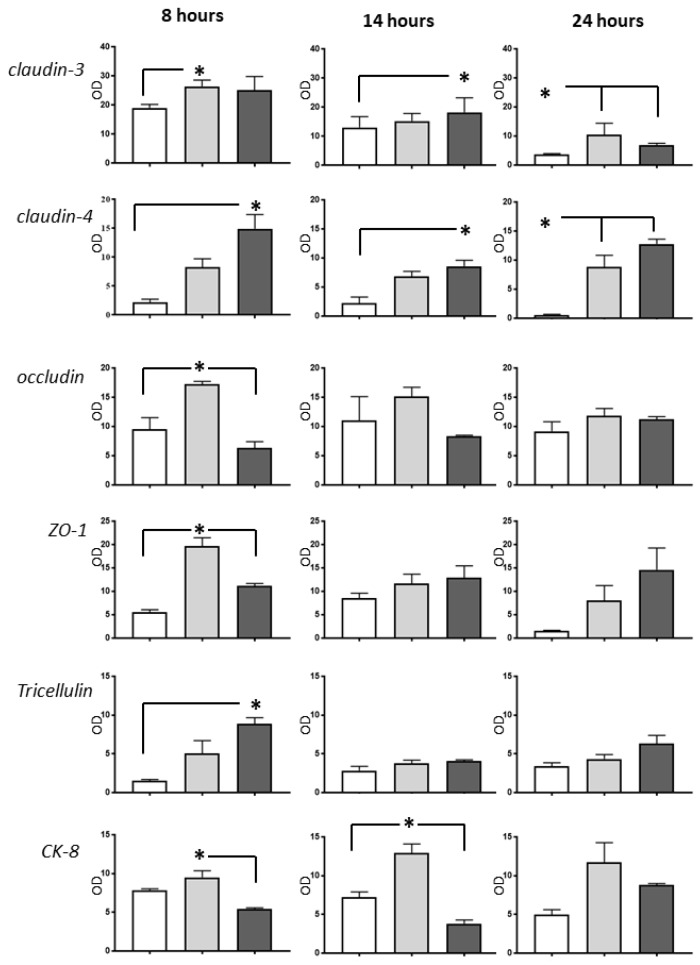
Western blot analysis of several junctional proteins and cytokeratin (CK)-8 in rat (white bars), pig (light grey bars), and human (dark grey bars) intestines after different periods of cold storage. Samples (15 µg) from the three species from the same time-point were run simultaneously (*n* = 3–4). Results (mean ± standard error) were normalized to glyceraldehyde-3-phosphate dehydrogenase and presented as semiquantitative results (optical density, OD); * *p* < 0.05.

Pig tissue expressed more occludin at eight hours as well as more Ck8 protein at all time points compared to rats (Figure 4). 

## 3. Discussion

Ischemia–reperfusion injury remains a major concern after intestinal transplantation as tissue damage may favor bacterial translocation and sepsis, anastomotic leaks, and intestinal graft edema with risk for abdominal compartment syndrome [2]. Moreover, an advanced ischemic injury may promote the later occurrence of graft fibrosis and graft dysmotility [14]. The susceptibility of the intestine to ischemic injury and the life-threatening complications it may lead to, continues to mandate a search for protective and therapeutic interventions. 

Although some studies infer a higher resilience of porcine intestines towards intestinal ischemia as compared to rats [15,16,17], to our knowledge this is the first direct comparison between these species. The sequential evaluation of the intestinal preservation injury in this study found a substantially different pattern of changes in human intestines compared with rats and pigs, both regarding the time course and the type of tissue damage. Rat and human intestines developed significant mucosal changes already after eight hours of CS whereas porcine intestines revealed near-normal epithelium after the same time span. Conversely, both the human and porcine intestines developed significant submucosal edema, a feature not observed in rodents. 

Goblet cells are critical for the integrity and repair of the intestinal epithelium and are considered a good marker of intestinal health [18]. GC mucus depletion occurs rapidly after the onset of intestinal ischemia [19]. In rats and humans, goblet cell count decreased during the CS compared to normal tissue, whereas this phenomenon was absent in the porcine ileum, which revealed a stable GC number throughout the experiment. Interestingly, control porcine tissue had less GC compared to both rats and humans. It is unclear whether this finding is intrinsic to (juvenile) pigs or it is the result of preoperative fasting as a decrease in amount and mucus content of GC count have been reported early and after fasting or weaning and in malnourished piglets [20]. Notably, the initial GC count seemed higher in the human intestines, while it is likely that the human organ donors did not receive any enteral nutrition during the day preceding the organ procurement either. 

Pigs developed mucosal alterations at the later time points compared to humans and rats. For example, the epithelial lesion recorded after 24 h (massive epithelial lifting, grade 3) in the pig intestines usually occurred between 8 and 14 h of CS in rat and human intestines. Part of the explanation for these interspecies differences may be the higher mucosal cellularity in pigs, leading to a higher TJ density. The lesser amount of tissue PMNs in pigs may also play a role as the hypoxic, stressed leukocytes could release its lytic enzymes in the surrounding tissue already before reperfusion [21,22]. The practical consequence of this finding is that preservation studies using pig intestines would require longer CS periods than in rats to attain a significant tissue injury. When designing experimental studies that mimic the human situation it may be prudent to adjust the ischemia time required to create a comparable injury to reflect the interspecies differences.

Caspase-3 activation and increased apoptosis has been reported earlier following CS of rat kidneys, livers, and intestines [23,24,25,26]. Similarly, we found abundant active caspase-3 in rat intestines; however, caspase-3 positive pig or human enterocytes were significantly fewer. This intriguing finding is difficult to explain considering that humans had very few caspase-3 positive enterocytes but it may also reflect interspecies differences. In another study, under similar conditions (60 min of intestinal ischemia and 120 min of reperfusion) 75% of rat Paneth cells entered apoptosis, whereas only 25% of the human Paneth cells were found apoptotic [27].

Ischemia and ATP depletion disrupt the actin cytoskeleton in various types of cells and tissues [28,29]. The actin cytoskeleton plays essential roles in the functional and structural integrity of the cells, including the structure and function of tight junctions. Thus, one suggested mechanism behind the TJ dysfunction is the strain on the TJs by the neighboring, contracting cells. Internalization of TJ proteins and TJ disassembly have been shown to occur rapidly after various stimuli, followed by TJ dysfunction and increased permeability [30]. Earlier studies revealed quantitative TJ protein changes during intestinal ischemia [31] yet the qualitative changes (i.e., cytoplasmic shift, altered membrane staining pattern) revealed by the immunofluorescence claudin-3 staining may be equally relevant. Herein, the progress of injury seems to have coincided with the occurrence of significant qualitative and quantitative alterations in claudin expression (particularly the TJ-sealing protein, claudin-3), whereas the expression of ZO-1 seems to have limited importance for injury development. In addition, it is tempting to speculate that the rapid TJ-protein alterations in the non-fasted rats, particularly tricellulin and ZO-1, may be also due to the mucosal exposure to the aggressive intestinal chyle containing bile acids, pancreatic enzymes combined with the depletion of the protective CG and mucus layer. 

An advantage of the current study is the systematic use of distal small intestine. The different digestive functions of the various intestinal segments are also reflected in the varying content of different junctional proteins [32]. Hence, we minimized the differences between different anatomical areas as responsible for the differences noted between various junctional proteins in different species. An inherent drawback of the study was the use of human intestines from brain dead organ donors. This setting was both necessary and relevant as it mirrored the clinical situation of intestinal procurement and preservation for transplantation. Nonetheless, this may have induced additional changes and differences compared to the young, healthy animals, as brain death induces both local and systemic inflammatory processes [33,34]. The relatively short period of brain death customarily encountered in Sweden may have limited the effect of donor inflammation on the intestine. Besides brain death, organ donors may also have been subjected to hemodynamic instability, cardiac arrest, trauma, or medical interventions (vasopressors, fluid resuscitation) that may potentially have affected the intestine. Whereas some of these factors were present in our human tissue donors, these factors not always preclude intestinal donation [35]. Discordant age between study subjects (young animals vs middle-aged organ donors) could also be regarded as another limitation. Though the current practice usually restricts the use of intestinal donors older than 50 [36], the impact of age on the development of intestinal preservation injury is yet unknown. Last but not least, the antibodies used in the study may have different affinity to different species. This implies that a higher protein expression detected on the Western blot does not always reflect its real tissue expression but rather shows the antibody–antigen affinity, which may differ between species.

In conclusion, this report provides the first direct comparison of the development of intestinal preservation injury in the rat, pig, and human at histological and molecular levels. The current results suggest that porcine intestines have a slower development of the tissue injury compared to human intestines, while rat intestines appear to have a faster injury development. These differences should be taken into account when designing experimental studies to allow meaningful endpoints and results. 

## 4. Materials and Methods

### 4.1. Animals, Surgery, and Sampling

Male, Sprague–Dawley rats (*n* = 7) aged around 3 months were purchased from Charles River (Sulzfeld, Germany), housed in the University animal quarters, and acclimatized for one week. The rats received rat chow and water ad libitum and were not fasted before surgery. The study followed the regulations outlined by the European Union (2010/63/EU) and was reviewed and approved by the Gothenburg committee of the Swedish Animal Welfare Agency (#135/07). Under 2.5% isoflurane anesthesia, the small intestine was perfused with and stored in ice-cold histidine–tryptophan–ketoglutarate solution (HTK, Custodiol^®^, Fresenius Kohler Chemie GmbH, Alsbach-Hähnlein, Germany) as described earlier [15]. The distal half (ileum) was resected and its ends were tightly ligated using silk 3/0. After 8 h, 14 h, and 24 h of CS 3 cm segments of ileum were sampled and stored in 4% buffered formalin or snap frozen. 

Landrace pigs of either sex (*n* = 7), weighing around 30 kg were purchased from a commercial supplier and housed individually at the Pius Branzeu Center in Timisoara. Animals were acclimatized for one week, fed once daily with standard pig diet and provided with water ad libitum. Food was withdrawn 24 h before surgery but animals’ unrestricted access to water was maintained. All experiments were reviewed and approved by the Ethics and Deontology Committee for Research on Animals of the University of Medicine and Pharmacy, Timisoara, Romania (13008/9 May 2013). Following premedication with ketamine (20 mg/kg; Pfizer Pharma GmbH, Germany), xylazine (2 mg/kg), and atropine (0.05 mg/kg), pigs were intubated and ventilated using a mixture of isoflurane and oxygen. Using a previously described approach [16] and following perfusion with 1.5 L HTK solution, the complete small intestine was then excised. In an ice basin on a backtable, the last meter of the ileum was resected, placed in ice-cold HTK solution, and sampled after 8 h, 14 h, and 24 h of CS. Samples were stored in 4% buffered formalin or snap frozen.

### 4.2. Human Organ Donors 

Ileal segments were obtained from seven deceased brain dead (DBD) multiorgan donors with an intensive care unit stay of less than four days. Donor median age was 48 years (range 14–63) (additional donor information is provided in Table S1). The donors (or next of kin) previously consented for tissue use for medical research. The use of human tissue in the study was reviewed and approved by the regional ethical review committee (Dnr 204-17). 

Organ retrieval was performed in the standard fashion using retrograde aortic perfusion with HTK solution and venous venting through the inferior vena cava. One meter of the distal small intestine (ileum) was resected immediately after the organ perfusion with HTK (3–8 L) and before any other organ was removed. Bowel ends were stapled off and the specimen was placed in an organ bag with cold HTK on ice. After 8 h, 14 h, and 24 h of CS a 10–15 cm ileal segment was removed with a stapler and samples were either placed in 4% formalin or snap frozen. 

### 4.3. Histology

#### 4.3.1. Light Microscopy

Formalin-fixed tissue was paraffinized, embedded, and cut into five-micron sections. Sections were stained with hematoxylin and eosin, and intestinal preservation injury was scored blinded by two experienced observers using the Chiu/Park score [37] on seven fields from three different sections. 

Mucus-filled goblet cells (GCs) in the intestinal villi were stained using Alcian Blue staining and counted in ten random fields at high magnification (×400) by a single observer blinded to the study design.

Apoptosis was studied on paraffin sections using immunostaining for active (cleaved) caspase-3 using a Warp Red Chromogen kit (Bio-Care Medical, Concord, CA) according to the manufacturer’s instructions. Briefly, after deparaffinization, rehydration, and antigen retrieval using citrate buffer (10 mM, pH 6.0), sections were blocked and then incubated with primary rabbit antibody against cleaved caspase-3 (1:100, #D175; Cell Signaling Technology, Danvers, MA) for 1 h at room temperature followed by incubation with an anti-rabbit probe, a rabbit alkaline phosphatase polymer and warp red chromogen. Nuclei were stained using Myers hematoxylin. Positively labeled enterocytes were counted on ten random fields at high magnification (×400) by a single observer. Polymorphonuclear neutrophils (PMN) were stained using the Naphtol AS-D chloroacetate esterase kit (Sigma Chemicals, St Louis, Mo) and counted in the villi on ten random fields at high magnification (×400).

#### 4.3.2. Immunofluorescence

Paraffin sections were deparaffinized and rehydrated, then antigen retrieval was performed (citrate buffer). After species-specific blocking, slides were incubated overnight at 4 °C with antibodies against zonula occludens (ZO-1; 1:100, Invitrogen AB, Lidingö, Sweden) and claudin-3 (1:100; Abcam, UK). Thereafter, slides were incubated with secondary antibody conjugated with Alexa 488 and Alexa 594 (1:200; Invitrogen). The sections were counterstained with 4′6′-diamidino-2-phenylindole, mounted with aqueous mounting medium (Vector Laboratories, Burlingame, CA, USA), and examined by fluorescence microscopy (Leica). Nuclei on the villi were also counted. Image acquisition and processing were performed using the Leica LAS software.

#### 4.3.3. Western Blot Analyses of Intestinal Mucosa

Western blot protein analysis was performed using whole tissue frozen specimens as described earlier [15). In brief, after electrophoresis and protein transfer on poly-vinyl-difluoride membranes, the membranes were blocked, then incubated overnight at 4 °C with primary antibody against claudin-3 (34-1700, Invitrogen AB, Lidingö, Sweden), claudin-4 (32-9400, Invitrogen AB), tricellulin (48-8400, Invitrogen AB), cytokeratin-8 (ab53708, Abcam, Cambridge, UK), ZO-1 (33-9100, Invitrogen AB), occludin (71-1500, Invitrogen AB), and the loading control glyceraldehyde-3-phosphate dehydrogenase (GAPDH, IMG-5143A, Imgenex, San Diego, CA). After repeated washings, a secondary antibody was applied for 1 h at room temperature and visualization was carried out using the chemoluminescent enzyme substrate CDP-Star (Tropix, Bedford, MA). The signal intensities of specific bands were detected and analyzed using a Chemidox XRS cooled charge-couple device camera and Quantity One software (BioRad Laboratories, Hercules, CA). GAPDH was used as loading control. For each sample, the optical density of primary antibody was normalized to GAPDH. Before re-probing with a new primary antibody, the membranes were incubated with stripping buffer (Re-Blot Plus Mild Solution 10×, Millipore, Temecula, CA, USA).

### 4.4. Statistical Analysis

Nonparametric methods were used for statistical comparisons. Statistical differences between independent groups were calculated using the Kruskal–Wallis test corrected for multiple comparisons using the Tukey test, followed by the Mann–Whitney U test (GraphPad Prism6; GraphPad Software, La Jolla, CA). Data are presented as median (range) unless otherwise stated. Results were considered as statistically significant at *p* < 0.05.

## Supplementary Materials

Supplementary materials can be found at https://www.mdpi.com/1422-0067/20/13/3135/s1.
