# Supplementary material for: Intestinal Preservation Injury: A Comparison Between Rat, Porcine and Human Intestines
Source: Int J Mol Sci. 2019 Jun 27;20(13):3135. doi: 10.3390/ijms20133135 (PMC6650817; doi:10.3390/ijms20133135)
Supplement: Supplementary file 1 [file ijms-20-03135-s001.pdf]

Supplementary Table S1. Characteristics and variables of the seven human organ donors.

Donor	COD	Age	BMI	Gender	NA dose (µg/kg/min)	ICU stay
1	Cardiac arrest/Cerebral anoxia	51	30.1	F	Yes—dose N/A	2
2	Cerebral trauma	13	22.9	F	0.057	2
3	Cardiac arrest/Cerebral anoxia	27	23.7	M	N/A	1
4	Cerebral trauma	46	21	M	0.14	2
5	Spontaneous intracerebral bleeding	53	22.2	F	Yes—dose N/A	3
6	Spontaneous intracerebral bleeding	62	23	F	0.02	2
7	Cardiac arrest/Cerebral anoxia	48	28	F	0.2	2

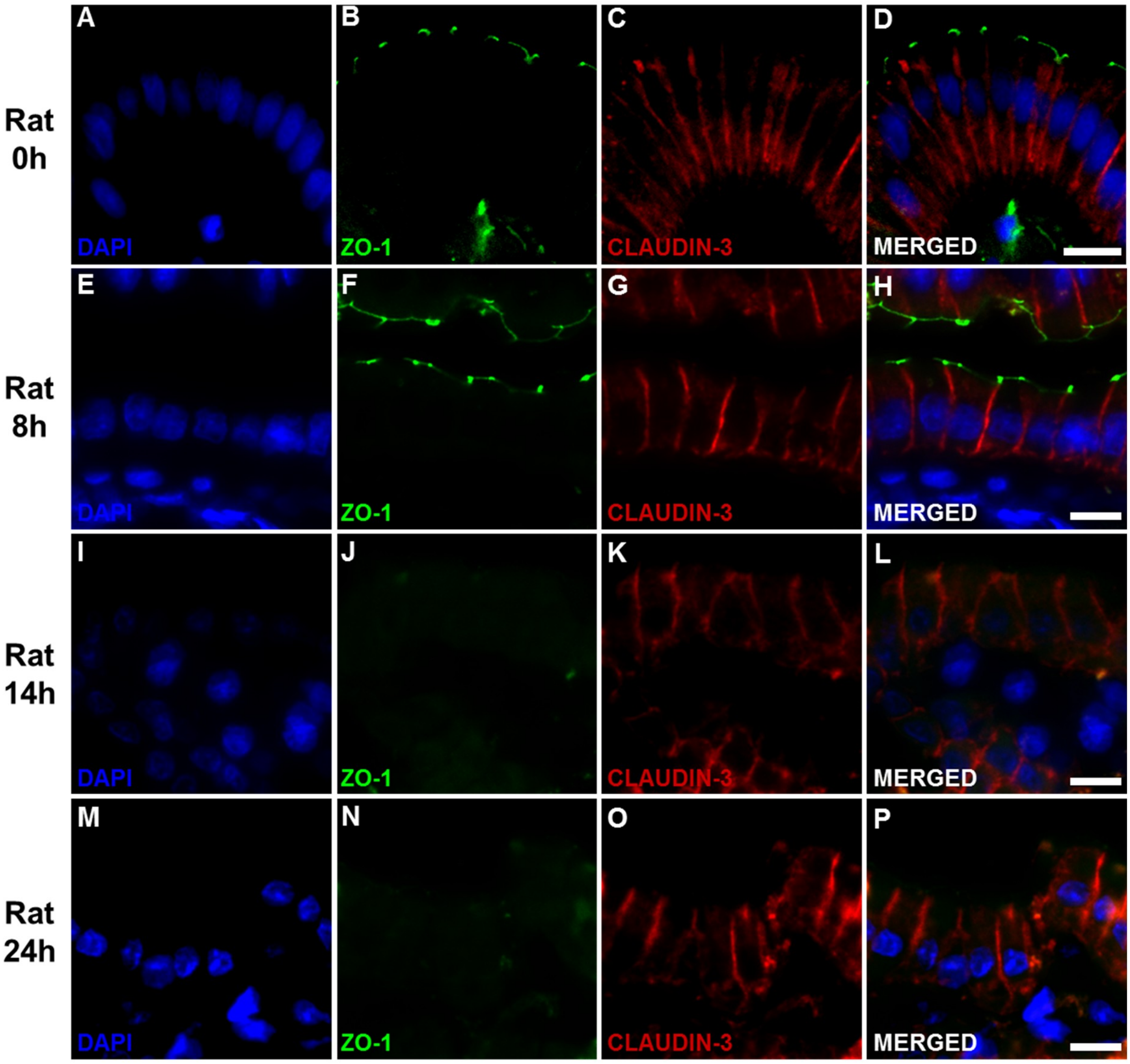

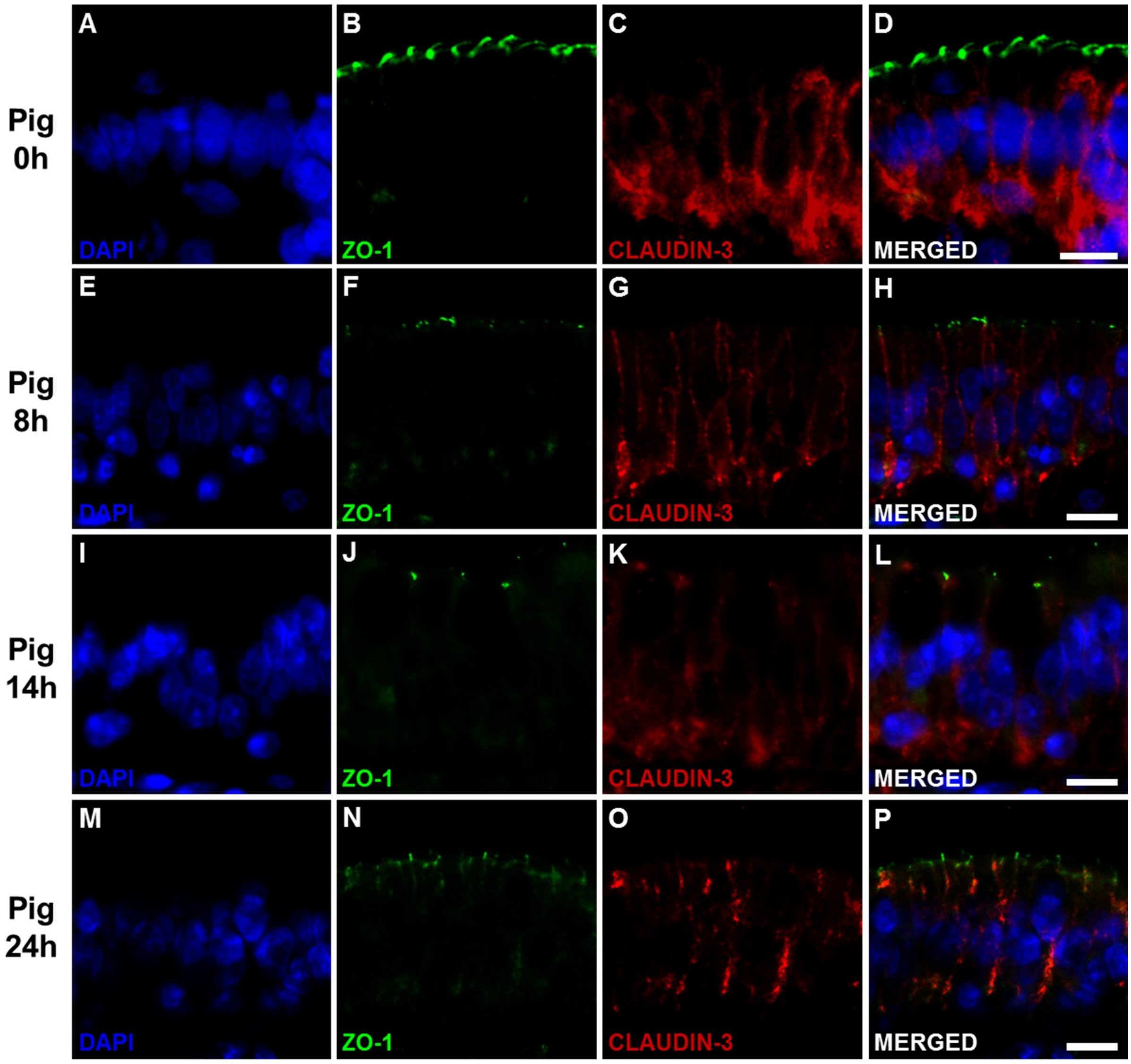
Pig 0h
Pig 8h
Pig 14h
Pig 24h
A
B
C
D
E
F
G
H
I
J
K
L
M
N
O
P
DAPI
ZO-1
CLAUDIN-3
MERGED

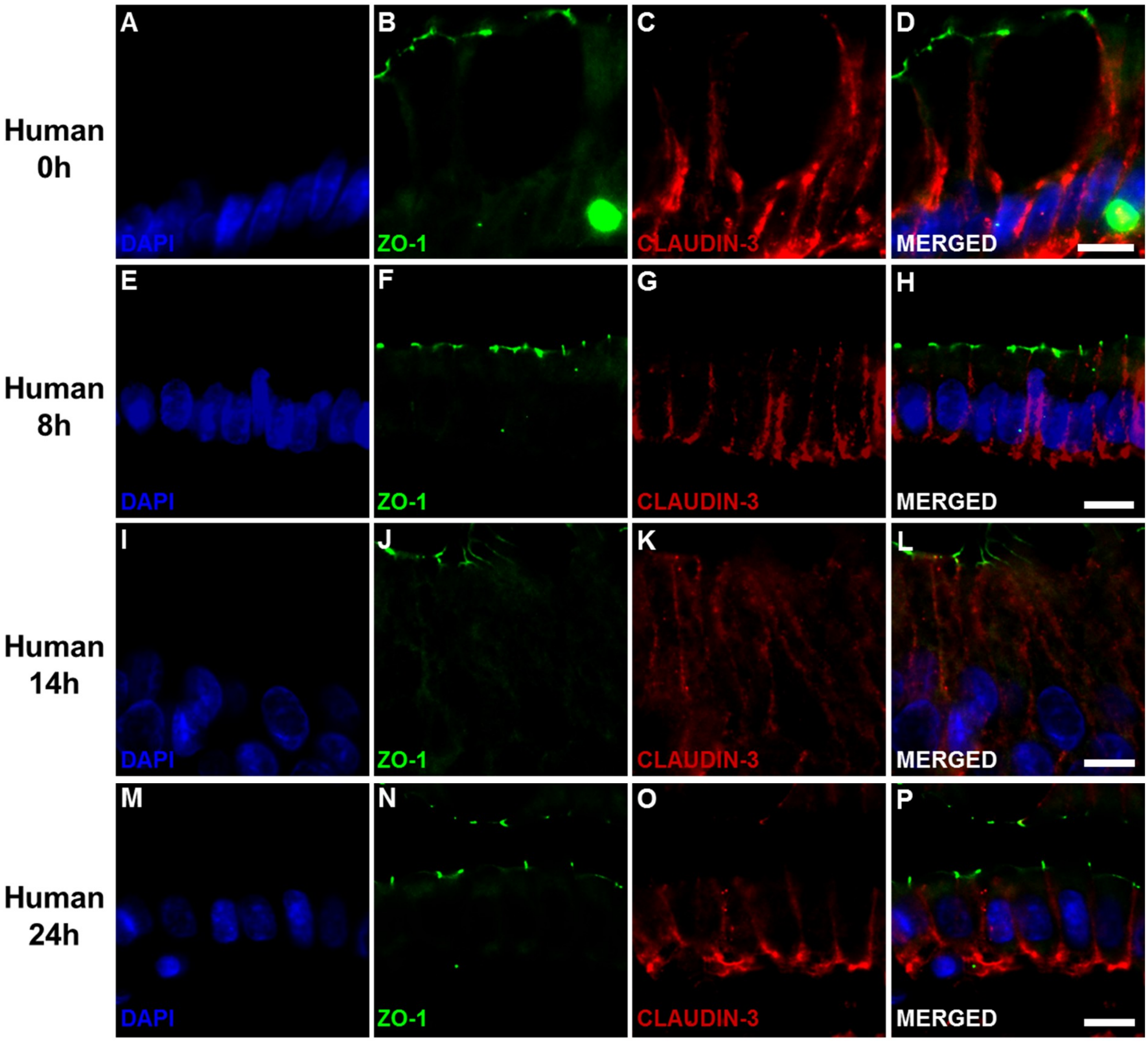

Supplementary Figure S1. Immunofluorescence staining for the tight junction proteins zonula occludens (ZO)-1 (green) and claudin-3 (red) in rat (first panel), pig (second panel), and human (third panel) intestine after eight hours (upper row), fourteen hours (middle row), and 24 hours of cold storage (lower row). Nuclei stained blue using 4',6-diamidino-2-phenylindole (DAPI). Original magnification, x400; scale bar, 10 μm.
